# Adjuvant postmastectomy radiotherapy might be associated with better survival in women with heart failure receiving total mastectomy

**DOI:** 10.1186/s13014-022-02000-x

**Published:** 2022-02-12

**Authors:** Jiaqiang Zhang, Shao-Yin Sum, Jeng-Guan Hsu, Ming-Feng Chiang, Tian-Shyug Lee, Szu-Yuan Wu

**Affiliations:** 1 Department of Anesthesiology and Perioperative Medicine, Henan Provincial People’s Hospital, People’s Hospital of Zhengzhou University, Zhengzhou, Henan China; 2 Department of General Surgery, Lo-Hsu Medical Foundation, Lotung Poh-Ai Hospital, Yilan, Taiwan; 3 Graduate Institute of Business Administration, Fu Jen Catholic University, Taipei, Taiwan; 4 Division of Gastroenterology and Hepatology, Department of Internal Medicine, Lo-Hsu Medical Foundation, Lotung Poh-Ai Hospital, Yilan, Taiwan; 5 Department of Food Nutrition and Health Biotechnology, College of Medical and Health Science, Asia University, Taichung, Taiwan; 6 Big Data & Cancer Center, Lo-Hsu Medical Foundation, Lotung Poh-Ai Hospital, Yilan, Taiwan; 7 Division of Radiation Oncology, Lo-Hsu Medical Foundation, Lotung Poh-Ai Hospital, No. 83, Nanchang St., Luodong Township, Yilan County 265 Taiwan; 8 Department of Healthcare Administration, College of Medical and Health Science, Asia University, Taichung, Taiwan; 9 Cancer Center, Lo-Hsu Medical Foundation, Lotung Poh-Ai Hospital, Yilan, Taiwan; 10 Centers for Regional Anesthesia and Pain Medicine, Taipei Municipal Wan Fang Hospital, Taipei Medical University, Taipei, Taiwan

**Keywords:** Breast cancer, Radiation-induced cardiovascular toxicity, Total mastectomy, Radiotherapy, Survival

## Abstract

**Background:**

To date, no data on the effect of adjuvant postmastectomy radiotherapy (PMRT) on oncologic outcomes, such as all-cause death, locoregional recurrence (LRR), and distant metastasis (DM), are available in women with left-side breast invasive ductal carcinoma (IDC) and heart failure with reduced ejection fraction (HFrEF).

**Patients and methods:**

We enrolled 646 women with left-breast IDC at clinical stages I–IIIC and HFrEF receiving radical total mastectomy (TM) followed by adjuvant PMRT or non-adjuvant PMRT. We categorized them into two groups based on their adjuvant PMRT status and compared their overall survival (OS), LRR, and DM outcomes. We calculated the propensity score and applied inverse probability of treatment weighting (IPTW) to create a pseudo-study cohort. Furthermore, we performed a multivariate analysis of the propensity score–weighted population to obtain hazard ratios (HRs).

**Results:**

In the IPTW-adjusted model, adjuvant PMRT (adjusted HR [aHR]: 0.52; 95% confidence interval [CI]: 0.37–0.74) was a significant independent prognostic factor for all-cause death (*P* = 0.0003), and the aHR (95% CI) of LRR and DM for adjuvant PMRT was 0.90 (0.79–0.96; *P* = 0.0356) and 0.89 (0.54–1.50; *P* = 0.6854), respectively, compared with the nonadjuvant PMRT group.

**Conclusion:**

Adjuvant PMRT was associated with a decrease in all-cause death, and LRR in women with left IDC and HFrEF compared with nonadjuvant PMRT.

## Key points

- **Question:** Is adjuvant postmastectomy radiotherapy (PMRT) worthy for women with left-side breast invasive ductal carcinoma (IDC) and heart failure with reduced ejection fraction (HFrEF) receiving total mastectomy (TM)?
- **Findings:** In the IPTW-adjusted models, adjuvant PMRT was associated with a decrease in all-cause death and LRR in women with left IDC and HFrEF compared with no adjuvant PMRT.
- **Meaning:** We suggest adjuvant PMRT for women with left-side IDC receiving TM, even when they have HFrEF.

## Introduction

Radiation-induced cardiovascular toxicity (RICT) is associated with a portion of the heart being placed in a radiation field [1]. For patients with left-sided breast cancers, careful treatment planning and usage of contemporary radiotherapy (RT) techniques are critical to minimize cardiac exposure to radiation [1]. Incidental irradiation dose to the heart as part of the initial treatment for breast cancer can result in a range of cardiotoxic effects, including coronary artery disease (CAD), cardiomyopathy, pericardial disease, valvular dysfunction, and conduction abnormalities [2–4]. At present, no recommended minimum radiation dose that is completely safe exists [3].

The association of RICT is not dependent on the presence or absence of a breast but on the volume of radiation to the heart [3, 4]. Thus, cardiotoxicities associated with RT differ between the postlumpectomy and postmastectomy settings; this is because in the postmastectomy setting, the RT field often includes the nodal tissues, and these nodes are not always targeted in the postlumpectomy setting [5, 6]. Thus, postmastectomy RT (PMRT) is more often associated with cardiac disease relative to postlumpectomy RT, but this is likely a result of the usually larger irradiated volumes of the heart in postmastectomy RT [5, 6]. Therefore, RICT in patients with breast cancer should be separately discussed by using different surgical techniques of breast-conserving surgery (BCS) and total mastectomy (TM). Hereby, we wanted to address the values of adjuvant PMRT for breast cancer patients with heart failure (HF) receiving TM with large RT field and high dose-volume to the normal heart.

The crucial issue is whether adjuvant PMRT can be safely given to women with HF and left-side breast cancer who receive TM. No data are available to address the value of adjuvant PMRT in women with breast cancer and HF receiving TM. HF due to left ventricle (LV) dysfunction is categorized according to LV ejection fraction (LVEF) as HF with reduced ejection fraction (LVEF ≤ 40%, known as HFrEF) [7–9]. Until now, no study has estimated the oncologic outcomes of adjuvant PMRT in women with breast invasive ductal carcinoma (IDC) and HFrEF receiving TM.

## Patients and methods

### Study population

In this cohort study, data were retrieved from the Taiwan Cancer Registry Database (TCRD). We enrolled women with HF with reduced ejection fraction (LVEF ≤ 40%; HFrEF) [7–9] who had received a diagnosis of left-side breast IDC between January 1, 2008, and December 31, 2018. The index date was the date of TM, and the follow-up duration was from the index date to December 31, 2019. The TCRD of the Collaboration Center of Health Information Application contains detailed cancer-related information of patients, including their clinical stage, pathologic stages, chemotherapy regimens, dose of chemotherapy, molecular status, drug use, hormone receptor status, radiation modalities and doses, and surgical procedures [10–13]. The study protocols were reviewed and approved by the Institutional Review Board of Tzu-Chi Medical Foundation (IRB109-015-B).

### Inclusion and exclusion criteria

The diagnoses of the enrolled patients with HFrEF were confirmed after their pathological data were reviewed, and the women with newly diagnosed left-side IDC were confirmed to have no other cancers or distant metastases. The women with HFrEF were included if they had received a left-side IDC diagnosis, were 20 years old or older, and had clinical stage I–IIIC (American Joint Committee on Cancer [AJCC], 8th edition) without metastasis. Patients with HFrEF were excluded if they had a history of cancer before the IDC diagnosis date, unknown pathologic types, missing sex data, unclear staging, or non-IDC histology. In addition, patients with unclear differentiation of tumor grade, missing HR status, missing data on hormone therapy or trastuzumab use, or unclear staging were excluded. Adjuvant treatments such as adjuvant chemotherapy, hormone therapy, or the trastuzumab use did not constitute exclusion criteria based on the National Comprehensive Cancer Network (NCCN) guidelines [14]. We also excluded patients with HFrEF with unclear data on surgical procedures such as TM or TM, ill-defined nodal surgery, unclear Charlson comorbidity index (CCI), or unclear differentiation from our cohort. Hormone receptor positivity was defined as ≥ 1% of tumor cells demonstrating positive nuclear staining through immunohistochemistry [15].

After applying the inclusion and exclusion criteria, we enrolled 646 women with HFrEF and AJCC clinical stage I–IIIC and left-side IDC who had received a TM followed by sentinel lymph node biopsy (SLNB) or axillary lymph node dissection (ALND) and divided them into two groups based on their adjuvant PMRT status to compare all-cause mortality: Group 1 (women with left-side IDC and HFrEF who received TM followed by adjuvant PMRT) and Group 2 (women with left-side IDC and HFrEF who received TM and no adjuvant PMRT). We also excluded women in Group 1 receiving nonstandard adjuvant PMRT. Standard postmastectomy RT is irradiation to both the chest wall and to the regional nodes with 50 Gy at least. These include the supraclavicular and infraclavicular nodes. We also include RT to the axilla except in some patients who underwent complete axillary dissection. Contemporary RT techniques were included in our study (intensity modulated radiation therapy [IMRT] and volumetric modulated arc therapy [VMAT]) and the conventional two-dimensional RT technique was excluded. The included contemporary RT techniques were three-dimensional RT and intensity-modulated radiation therapy. The incidence of comorbidities was scored using the CCI [16, 17]. Hypertension, diabetes, and coronary arterial diseases (CAD) were excluded from the CCI scores to avoid repetitive adjustment in multivariate analysis. Only comorbidities observed within 6 months before the index date were included; they were coded and classified according to the *International Classification of Diseases, 10th Revision, Clinical Modification* (ICD-10-CM) codes at the first admission or based on more than two repetitions of a code issued at outpatient department visits.

### Study covariates and statistical analysis

Significant independent predictors, namely age, diagnosis year, CCI score, differentiation, pT, pN, hypertension, CAD, diabetes, chemotherapy with anthracycline-based regimen, hormone receptor status, trastuzumab use, nodal surgery, and hospital level (academic or nonacademic), were analyzed using a multivariate analysis of the propensity score–weighted population to determine hazard ratios (HRs). We calculated the propensity score and applied inverse probability of treatment weighting (IPTW) to create a pseudo-study cohort; the weighted cohort avoids covariate bias and mimics randomized adjuvant PMRT or no adjuvant PMRT assignment: IPTW for patients with PMRT = 1/p(PMRT); IPTW for patients without PMRT = 1/(1 − p[PMRT]) [18, 19]. The independent predictors were examined in multivariable analyses after IPTW adjustment. Moreover, they were controlled for and were stratified in the analysis. The endpoint was all-cause death in the women with left-side IDC and HFrEF who received TM followed by adjuvant PMRT (Group 1, case group) and in the women with left IDC and HFrEF who received TM and had no adjuvant PMRT (Group 2, control group).

The cumulative incidence of death was estimated using the Kaplan–Meier method, and differences in the overall survival (OS), locoregional recurrence (LRR)–free survival, and distant metastasis (DM)–free survival between women with left IDC and HFrEF receiving TM followed by adjuvant PMRT versus no adjuvant PMRT were determined using a log-rank test. After confounders were adjusted for, IPTW-adjusted models were used to determine the time from the index date to all-cause mortality in the women with left IDC and HFrEF who received TM followed by adjuvant PMRT or no adjuvant PMRT. Subsequently, in a multivariate analysis, HRs were adjusted for age, diagnosis year, CCI scores, differentiation, pT, pN, hypertension, CAD, diabetes, chemotherapy with anthracycline, hormone receptor status, trastuzumab use, nodal surgery, and hospital levels. All analyses were conducted using SAS (Version 9.4; SAS, Cary, NC, USA), and a two-tailed *P* value < 0.05 was considered statistically significant.

## Results

### Study cohort

We enrolled 646 women with left-breast IDC at clinical stages I–IIIC and HFrEF who received TM followed by adjuvant PMRT or no adjuvant PMRT (Table 1). Among these women, 143 with left IDC and HFrEF received TM followed by adjuvant PMRT (Group 1) and 503 with left IDC and HFrEF received TM with no adjuvant PMRT (Group 2). After IPTW was executed using the propensity score, the covariates between Groups 1 and 2 were found to be homogenous. The median follow-up durations after the index date were 6.96 and 5.09 years for women with left IDC and HFrEF who received TM followed by adjuvant PMRT or no adjuvant PMRT, respectively. All standardized differences in covariates were smaller than 0.1 (Table 1) and were homogenous between the two groups [20].

**Table 1:** Demographics of patients with breast cancer and heart failure with reduced ejection fraction who received total mastectomy in the propensity score–weighted population through inverse probability of treatment weighting

	Propensity score weighting population				
	Adjuvant RT N = 143		Non-RT N = 503		Standardized difference
	n	(%)	n	(%)	
Age					
Mean (SD)	67.5	(11.3)	67.7	(11.2)	0.0215
Median (Q1–Q3)	67	(57–72)	67	(61–77)	
20–69	67	(46.9)	238	(47.9)	0.0096
70+	76	(53.1)	259	(52.1)	
Diagnosis year					
2009–2012	88	(50.9)	250	(50.3)	0.0115
2013–2016	85	(49.1)	247	(49.7)	
CCI scores					
0	44	(30.8)	123	(24.5)	0.1433
1+	99	(69.2)	380	(75.5)	
Differentiation					
I	20	(14.0)	72	(14.3)	0.0212
II	77	(53.8)	273	(54.3)	0.0311
III	46	(32.1)	158	(31.4)	0.0207
AJCC pathologic stage					
II	17	(12.8)	61	(12.1)	0.0114
IIIA	68	(47.6)	241	(47.9)	0.0162
IIIB–C	58	(40.6)	201	(40.0)	0.0142
pT					
pT1	9	(6.3)	30	(5.9)	0.0238
pT2	58	(40.6)	202	(40.2)	0.0149
pT3–4	76	(53.1)	271	(53.9)	0.0130
pN					
pN0	57	(39.8)	200	(39.8)	0.0000
pN1	43	(30.1)	151	(30.0)	0.0013
pN2–3	43	(30.1)	152	(30.2)	0.0001
Hypertension	110	(76.9)	387	(76.9)	0.0001
CAD	53	(37.1)	184	(36.6)	0.0092
Diabetes	60	(42.0)	215	(42.7)	0.0097
Anthracycline-based chemotherapy	76	(53.1)	260	(51.7)	0.0280
Hormone receptor positive	59	(41.3)	229	(45.5)	0.0934
Trastuzumab use	69	(48.3)	246	(48.9)	0.0031
Nodal surgery					
ALND	87	(60.8)	302	(60.0)	0.0079
SLNB	56	(39.2)	201	(40.0)	
Hospital level					
Academic center	80	(55.9)	285	(56.7)	0.0181
Nonacademic center	63	(44.1)	218	(43.3)	

*RT* radiotherapy, *IQR* interquartile range, *SD* standard deviation, *AJCC* American Joint Committee on Cancer, *HER2* human epidermal growth factor receptor-2, *CCI* Charlson comorbidity index, *T* tumor, *N* nodal, *pT* pathologic tumor stage, *pN* pathologic nodal stage, *ALND* axillary lymph node dissection, *SNLB* sentinel lymph node biopsy, *CAD* coronary arterial disease

### Effects of adjuvant PMRT on oncologic outcomes in women with left-side IDC and HFrEF receiving TM

IPTW-adjusted models indicated that adjuvant PMRT was a significantly better independent prognostic factor for OS, and LRR in the women with left IDC and HFrEF receiving TM (Table 2). Adjuvant PMRT (adjusted HR [aHR]: 0.52; 95% confidence interval [CI]: 0.37–0.74) was a significant independent prognostic factor for all-cause death (*P* = 0.0003; Table 2). In the IPTW-adjusted model, the aHR (95% CI) for LRR in the adjuvant PMRT group was 0.90 (0.79–0.96; *P* = 0.0356; Table 2) compared with the no adjuvant PMRT group. Moreover, the aHR (95% CIs) for DM in the adjuvant PMRT group was 0.89 (0.54–1.50; *P* = 0.6854) compared with the no adjuvant PMRT group (Table 2).

**Table 2:** Multivariate analysis of propensity score–weighted population with breast cancer and heart failure with reduced ejection fraction receiving total mastectomy

	Death			Local recurrence			Distant metastasis		
	aHR*	(95% CI)	*P* value	aHR*	(95% CI)	*P* value	aHR*	(95% CI)	*p* value
Adjuvant RT									
No	Ref		0.0003	Ref		0.0356	Ref		0.6854
Yes	0.52	(0.37–0.74)		0.90	(0.79–0.96)		0.89	(0.54–1.50)	
Age									
20–69	Ref		0.0020	Ref		0.1200	Ref		0.2901
70+	1.63	(1.20–2.22)		1.29	(0.81–2.49)		0.75	(0.44–1.28)	
Diagnosis year									
2009–2012	Ref		0.3507	Ref		0.2770	Ref		0.7421
2013–2016	0.79	(0.69–1.31)		0.73	(0.58–1.21)		0.81	(0.57–1.75)	
CCI scores									
0	Ref		0.0322	Ref		0.1434	Ref		0.2112
1	1.26	(1.14–1.97)		1.21	(0.92–1.79)		1.35	(0.85–1.97)	
Differentiation									
I	Ref		0.0177	Ref		0.0146	Ref		0.0046
II	1.09	(1.01–1.60)		1.36	(1.02–3.59)		1.36	(1.02–3.59)	
III	1.47	(1.09–2.40)		1.37	(1.11–3.71)		1.37	(1.01–3.71)	
pT									
pT1	Ref		< 0.0001	Ref		0.0016	Ref		0.0196
pT2	1.38	(1.07–1.97)		1.35	(1.05–3.12)		1.09	(1.04–3.04)	
pT3–4	2.91	(1.90–4.44)		2.62	(1.19–4.72)		2.35	(1.13–4.89)	
pN									
pN0	Ref		< 0.0001	Ref		0.0040	Ref		0.0082
pN1	1.94	(1.38–2.72)		1.09	(1.03–1.41)		2.38	(1.37–4.12)	
pN2–3	2.90	(2.01–4.18)		1.26	(1.06–1.37)		1.88	(1.01–3.51)	
Hypertension	1.08	(0.77–1.81)	0.4882	0.90	(0.59–1.56)	0.7217	0.95	(0.69–1.48)	0.8021
CAD	1.11	(0.71–1.92)	0.3427	0.84	(0.53–1.39)	0.6914	0.94	(0.78–1.59)	0.3426
Diabetes	1.11	(0.73–1.90)	0.3422	1.01	(0.70–1.51)	0.4521	0.90	(0.55–1.91)	0.8909
Hormone receptor positive	0.87	(0.80–0.91)	0.0312	0.77	(0.45–0.82)	0.0204	0.72	(0.63–0.97)	0.0322
Trastuzumab use	1.07	(0.87–1.42)	0.34661	1.09	(0.58–2.01)	0.3831	1.06	(0.81–1.54)	0.3421
Anthracycline-based chemotherapy	0.94	(0.57–1.88)	0.4065	0.93	(0.78–1.83)	0.2412	0.84	(0.70–2.20)	0.1683
Nodal surgery									
ALND	Ref		0.3322	Ref		0.2434	Ref		0.2112
SLNB	1.06	(0.54–1.09)		1.01	(0.82–1.79)		1.15	(0.85–2.97)	
Hospital level									
Academic center	Ref		0.2177	Ref		0.2177	Ref		0.8146
Nonacademic center	0.99	(0.61–1.60)		0.99	(0.61–1.60)		1.36	(0.52–3.59)	

*aHR* adjusted hazard ratios, *CIs* confidence intervals, *HR* hormone receptor, *Her-2* human epidermal growth factor receptor-2, *CCI* Charlson comorbidity index, *T* tumor, *N* nodal, *pT* pathologic tumor stage, *pN* pathologic nodal stage, *ALND* axillary lymph node dissection, *SNLB* sentinel lymph node biopsy, *ref* reference group, *RT* radiotherapy

*All covariates mentioned in Table were adjusted

### Other independent predictors of all-cause death, LRR, and DM in the women with left IDC and HFrEF receiving TM

Old age (> 70 years), CCI ≥ 1, advanced pT stages (pT2–4), advanced pN stages (pN1–3), hormone receptor negative status, and differentiation Grade II and III were identified as crucial independent poor prognostic factors for OS (Table 2). IPTW-adjusted models were adjusted for age, diagnosis year, CCI score, differentiation, pT, pN, hypertension, CAD, diabetes, chemotherapy with anthracycline-based regimen, hormone receptor status, trastuzumab use, nodal surgery, and hospital levels; the aHRs (95% CIs) of all-cause death for age > 70 years, CCI ≥ 1, differentiation Grades II and III, pT2, pT3–4, pN1, pN2–3, and hormone receptor positive status were 1.63 (1.20–2.22), 1.26 (1.14–1.97), 1.09 (1.01–1.60), 1.47 (1.09–2.40), 1.38 (1.07–1.97), 2.91 (1.90–4.44), 1.94 (1.38–2.72), 2.90 (2.01–4.18), and 0.87 (0.80–0.91) compared with age 20–70 years, CCI = 0, differentiation grade I, pT1, pN0, and hormone receptor negative status, respectively (Table 2). IPTW-adjusted models also revealed the aHRs (95% CIs) of LRR for differentiation grade II, differentiation grade III, pT2, pT3–4, pN1, pN2–3, and hormone receptor positive status to be 1.36 (1.02–3.59), 1.37 (1.11–3.71), 1.35 (1.05–3.12), 2.62 (1.19–4.72), 1.09 (1.03–1.41), 1.26 1.06–1.37), and 0.77 (0.45–0.82) compared with differentiation grade I, pT1, pN0, and hormone receptor negative status, respectively. Moreover, the aHRs (95% CIs) of DM for differentiation grade II, differentiation grade III, pT2, pT3–4, pN1, pN2–3, and hormone receptor positive status were 1.36 (1.02–3.59), 1.37 (1.01–3.71), 1.09 (1.04–3.04), 2.35 (1.13–4.89), 2.38 (1.37–4.12), 1.88 (1.01–3.51), and 0.72 (0.63–0.97) compared with differentiation grade I, pT1, pN0, and hormone receptor negative status, respectively.

### Survival curves of adjuvant PMRT or no adjuvant PMRT in women with left IDC and HFrEF receiving TM

**Fig. 1:**
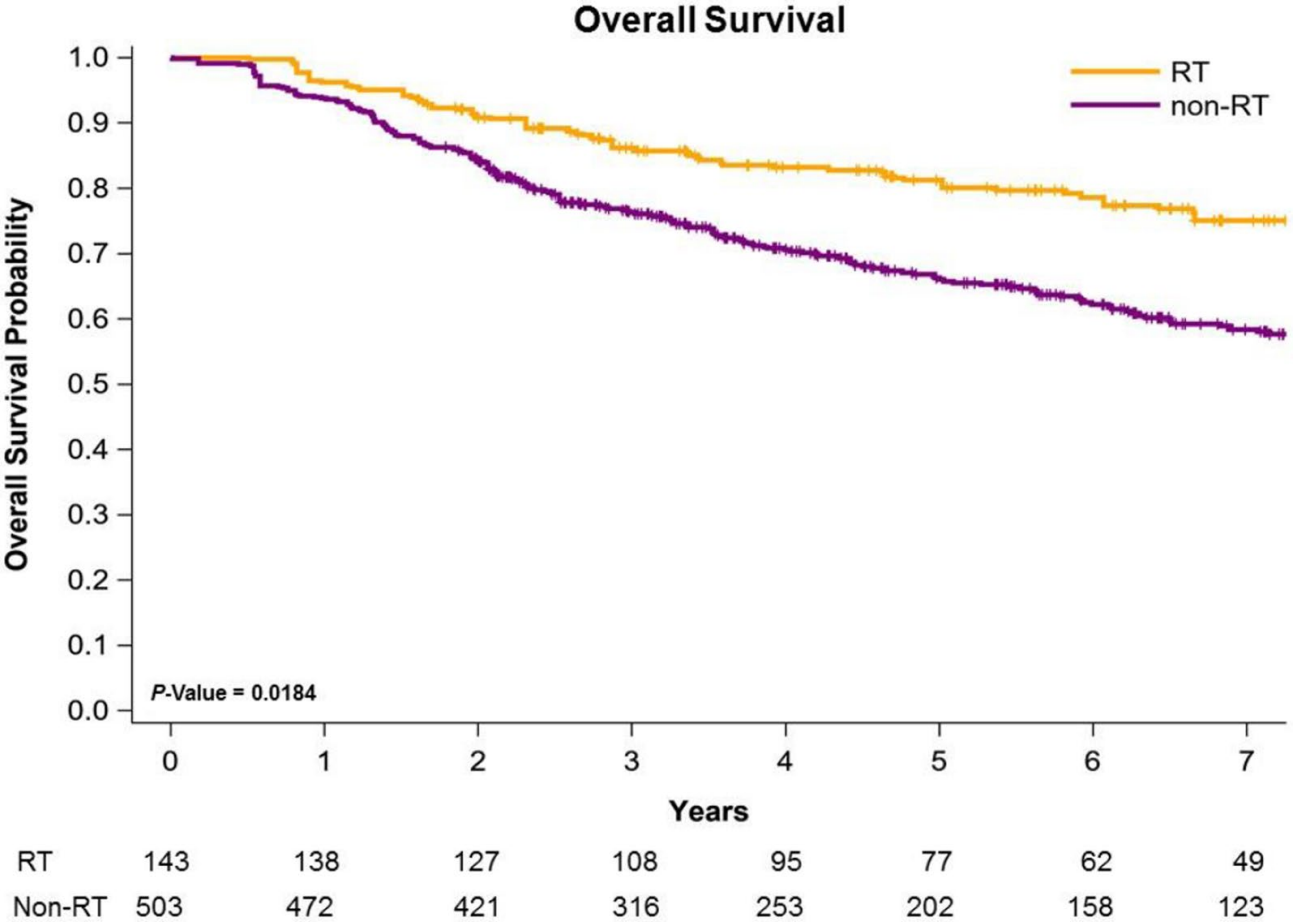
Kaplan–Meier overall survival curves of propensity score–weighted population with breast cancer and heart failure with reduced ejection fraction receiving total mastectomy

**Fig. 2:**
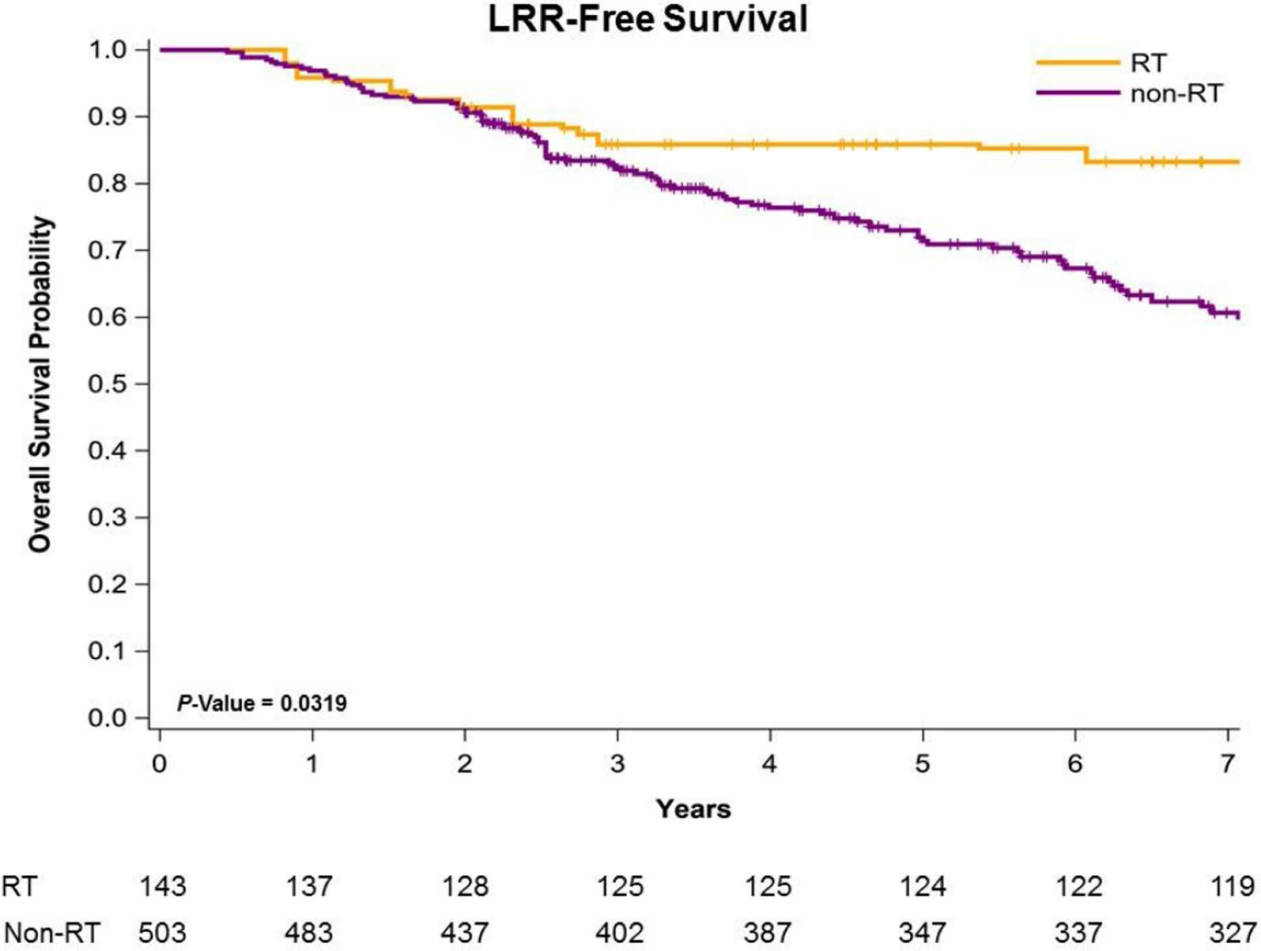
Kaplan–Meier locoregional recurrence-free survival curves of propensity score–weighted population with breast cancer and heart failure with reduced ejection fraction receiving total mastectomy

**Fig. 3:**
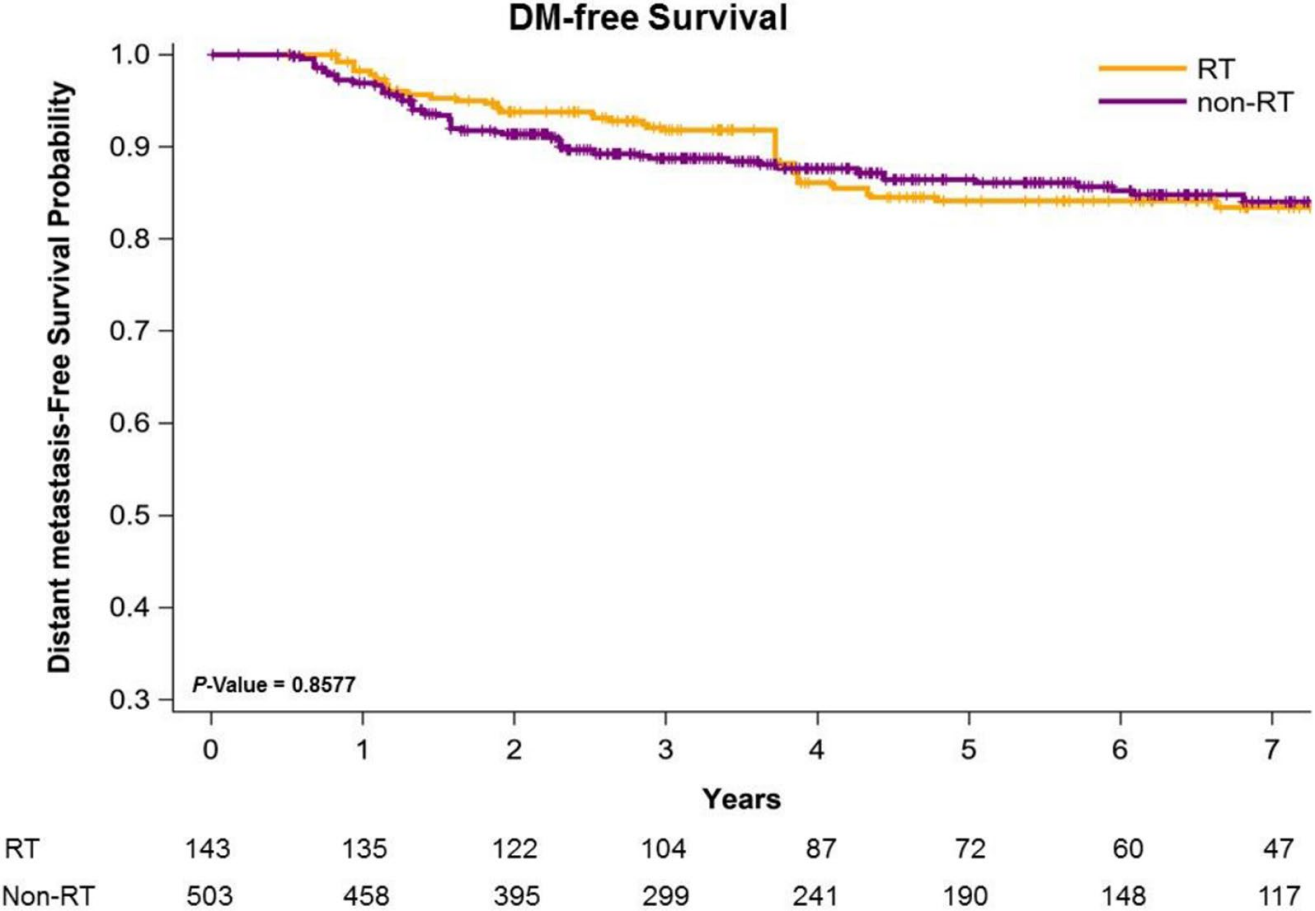
Kaplan–Meier distant metastasis–free survival curves of propensity score–weighted population with breast cancer and heart failure with reduced ejection fraction receiving total mastectomy

Figure 1 presents Kaplan–Meier curves that illustrate the OS of the women with left IDC and HFrEF receiving TM with adjuvant PMRT or no adjuvant PMRT. The 5-year overall survival rates were 86.01% and 67.32% in the adjuvant PMRT and no adjuvant PMRT groups, respectively (Fig. 1); the OS rate was associated with an increasing in the adjuvant PMRT group (log-rank test, *P* = 0.0184) compared with the non-RT group. Additionally, the 5-year LRR-free survival in women with left IDC and HFrEF receiving TM was 88.43% and 73.65% in the adjuvant PMRT group and no adjuvant PMRT group, respectively (Fig. 2; log-rank test,* P* = 0.0319). The 5-year DM-free survival in women with left IDC and HFrEF receiving TM was 84.43% and 86.91% in the adjuvant PMRT group and no adjuvant PMRT group, respectively (Fig. 3; log-rank test, *P* = 0.8577).

## Discussion

PMRT has two potential benefits: one is a decrease in the rate of locoregional recurrence, and another is an increase in long-term breast cancer-specific and overall survivals for certain patient populations. These benefits have been consistently reported in multiple studies [21–23]. Decisions on who should receive PMRT depend on the baseline risk for recurrence. The success of RT, used either alone or in combination with other modalities, has resulted in large cohorts of breast cancer survivors who are vulnerable to late complications such as RICT from RT [5, 24–30]. Numerous treatment-related factors are responsible for cardiotoxicity in women with breast cancer [31–41]. Thus, we conducted the study to determine the survival benefits offered by adjuvant PMRT in women with left-side IDC and HFrEF receiving TM.

Patients with breast cancer might experience adverse effects from many cardiotoxic treatments such as adjuvant PMRT, anthracycline-based chemotherapy, or trastuzumab [5, 6, 24–41]. Although cardiovascular diseases such as HF, heart attacks, and stroke remain the leading cause of death in women, many believe breast cancer to be more deadly [42]. In fact, the risk of RICT should be weighed against the potential benefits of adjuvant PMRT with respect to the patients’ prognosis and likely clinical benefit [5, 24–30]. Until now, no data have been available for the evaluation of oncologic outcomes (OS, LRR, and DM) of adjuvant PMRT in women with left-side breast IDC and HFrEF receiving TM. This is the first study to explore the value of adjuvant PMRT for women with left-side breast IDC and HFrEF receiving TM. As shown in Table 2, adjuvant PMRT resulted in better OS, and LRR-free status compared with no adjuvant PMRT in women with left-side breast IDC and HFrEF receiving TM. The potential reasons might be the recent decline in mortality in women with HF [43, 44] and the advances in contemporary RT techniques with reduced irradiation volumes to the heart [2, 26, 27].

According to our literature review, this is the first study to estimate the oncologic outcomes of adjuvant PMRT among women with left-side breast IDC and HFrEF receiving TM. No consensus or evidence for the use of adjuvant PMRT in women with left-side breast IDC and HFrEF receiving TM is present. In the IPTW-adjusted models, adjuvant PMRT was associated with a decrease in the risk of all-cause death, and LRR among women with left-side breast IDC and HFrEF receiving TM (Table 2). The improvement in contemporary RT techniques with decreased irradiation doses and decreased volumes to the heart and the long-term improvement in mortality rates in patients with HFrEF over time might have caused the significant beneficial oncologic outcomes of adjuvant PMRT in women with left-side breast IDC and HFrEF receiving TM [2, 26, 27]. Our study is the first to demonstrate that the potential benefits of adjuvant PMRT with contemporary RT techniques outweigh the risk of RICT given the patients' prognosis and likely long-term OS and LRR benefits (Table 2). According to our findings, we strongly suggested that women with left-side breast IDC and HFrEF receiving TM should also receive adjuvant PMRT to decrease the risk of all-cause death, and LRR.

As shown in Table 2, adjuvant PMRT was a significant prognostic factor for OS and LRR compared with no adjuvant PMRT in women with left-side IDC and HFrEF receiving TM; moreover, old age (> 70 years), CCI ≥ 1, advanced pT stages (pT2–4), advanced pN stages (pN1–3), hormone receptor negative status, and differentiation Grade II–III were significant prognostic factors for OS, compatible with findings of previous studies [10, 11, 45–52]. Moreover, advanced pT stages (pT2-4), pN stages (pN1–3), hormone receptor negative status, and differentiation Grade II–III were significant poor prognostic factors for LRR and DM in women with left-side breast IDC and HFrEF receiving TM, which is also compatible with findings of previous studies [10, 11, 45–52]. Our findings of prognostic factors for OS, LRR, and DM in women with IDC and HFrEF receiving TM are similar with those of previous studies [10, 11, 45–52], and no additional prognostic factor has been identified in previous studies other than the ones determined in the current study irrespective of whether underlying HFrEF was present.

The potential reasons of better oncologic outcomes on adjuvant PMRT for breast IDC with HFrEF might be attributed to the modern RT techniques. The use of modern RT techniques (such as IMRT and VMAT) as well as the reduction of treatment volumes (partial breast irradiation) allow to reduce acute and late side effects [53–55]. The contemporary RT techniques allow more precise RT field to target volume and decrease RT dose-volume to heart contributed to less RICT [53, 54]. Therefore, breast IDC patients with HFrEF receiving TM and adjuvant PMRT could get benefits from PMRT and less acute and late toxicity to heart contributed to better oncologic outcomes like OS and LRR-free survival (Table 2, Figs. 1, 2).

A strength of our study was that it was the first cohort study to estimate the survival outcomes of adjuvant PMRT or no adjuvant PMRT among women with left-side IDC and HFrEF receiving TM. The covariates between the adjuvant PMRT and no adjuvant PMRT groups were homogenous for women with left-side IDC and HFrEF receiving TM, with no selection bias (Table 1). No study has estimated the effect of adjuvant PMRT on women with left-side IDC and HFrEF receiving TM. In our study, the poor prognostic factors for OS in women with left-side IDC and HFrEF receiving TM were old age, CCI ≥ 1, advanced pT stages (pT2–4), advanced pN stages (pN1–3), hormone receptor negative status, and differentiation Grade II–III of (Table 2), which are consistent with factors in women with breast cancer without HFrEF reported in previous studies [48–52]. Furthermore, our study is the first to demonstrate the benefits of adjuvant PMRT with contemporary RT techniques for OS, LRR, and DM in women with left-side IDC and HFrEF receiving TM. Our findings should be considered in future clinical practice and prospective clinical trials of HF and RT for breast cancer. We suggest that adjuvant PMRT is valuable to achieving better outcomes of OS, LRR, and DM in women with left-side IDC and HFrEF receiving TM.

This study has some limitations. First, because all women with left-side breast IDC and HFrEF were enrolled from an Asian population, the corresponding ethnic susceptibility compared with the non-Asian population remains unclear; hence, our results should be cautiously extrapolated to non-Asian populations. However, no evidence exists as to the differences in oncologic outcomes in Asian versus non-Asian patients with breast IDC and HFrEF receiving TM. Second, a weak point of the study that the median follow up (6.96 and 5.09 years) could be too short for evaluation the impact on breast cancer survival or late heart side effects. Third, the diagnoses of all comorbid conditions were based on ICD-10-CM codes. However, the combination of Taiwanese TCRD and National Health Insurance Research Database (NHIRD) data appears to be a valid resource for population research on cardiovascular diseases, stroke, or chronic comorbidities [56–58]. Moreover, the Taiwan Cancer Registry Administration randomly reviews charts and interviews patients to verify the accuracy of the diagnoses, and hospitals with outlier chargers or practices may be audited and subsequently be heavily penalized if any malpractice or discrepancy is detected. Accordingly, to obtain crucial information on population specificity and disease occurrence, a large-scale randomized trial comparing carefully selected patients undergoing suitable treatments is essential. Finally, the TCRD does not contain information regarding dietary habits or body mass index, which may be risk factors for mortality. Nevertheless, considering the magnitude and statistical significance of the observed effects in this study, these limitations are unlikely to affect the conclusions.

## Conclusions

Adjuvant PMRT was associated with a decrease in all-cause death and LRR among women with left-side breast IDC and HFrEF compared with no adjuvant PMRT. We suggest adjuvant PMRT for women with left-side IDC receiving TM, even if they have HFrEF.

## Data Availability

The data sets supporting the study conclusions are included in this manuscript and its supplementary files. **For software**: Project name: not applicable; Project homepage: not applicable; Archived version: not applicable; Operating system(s): not applicable; Programming language: not applicable; Other requirements: not applicable; License: not applicable; Any restrictions for use by nonacademicians: not applicable.

## Abbreviations

PMRT: Postmastectomy RT
LRR: Locoregional recurrence
DM: Distant metastasis
IDC: Invasive ductal carcinoma
HFrEF: Heart failure with reduced ejection fraction
TM: Total mastectomy
OS: Overall survival
aHR: Adjusted hazard ratio
HR: Hazard ratio
IPTW: Inverse probability of treatment weighting
CI: Confidence interval
AJCC: American Joint Committee on Cancer
TCRD: Taiwan Cancer Registry Database
SD: Standard deviation
HER2: Human epidermal growth factor receptor-2
SLNB: Sentinel lymph node biopsy
ALND: Axillary lymph node dissection
CCI: Charlson comorbidity index
ICD-10-CM: International Classification of Diseases, 10th Revision, Clinical Modification
NCCN: National Comprehensive Cancer Network
RT: Radiotherapy
RICT: Radiotherapy-related cardiotoxicity
TM: Total mastectomy
HF: Heart failure
LV: Left ventricular
LVEF: Left ventricular ejection fraction
T: Tumor
N: Nodal
pT: Pathologic tumor stage
pN: Pathologic nodal stage
NHIRD: National Health Insurance Research Database
